# Supplementary material for: Awareness of anthrax disease and the knowledge of its transmission and symtoms identification: A cross sectional study among butchers in Ile-Ife
Source: PLOS Glob Public Health. 2026 Mar 20;6(3):e0005387. doi: 10.1371/journal.pgph.0005387 (PMC13004521; doi:10.1371/journal.pgph.0005387)
Supplement: S1 Table — (DOCX) [file pgph.0005387.s001.docx]

S1 Table: Sociodemographic Variables

| Variable | Legend |
| --- | --- |
| age | Age of participant (in years) |
| education | Education level: 1 = Primary, 2 = Secondary, 3 = Tertiary, 4= Postgraduate |
| religion | Religion: 1 = Christian, 2 = Muslim 3= traditional |
| tribe | Tribe/Ethnicity: 1 = Yoruba, 2 = Hausa, 3= Igbo |
| income | Monthly income (in naira) |
| gender | Gender: 1 = Male, 2 = Female |
