# Supplementary material for: Awareness of anthrax disease and the knowledge of its transmission and symtoms identification: A cross sectional study among butchers in Ile-Ife
Source: PLOS Glob Public Health. 2026 Mar 20;6(3):e0005387. doi: 10.1371/journal.pgph.0005387 (PMC13004521; doi:10.1371/journal.pgph.0005387)
Supplement: S2 Table — (DOCX) [file pgph.0005387.s002.docx]

S2 Table: Animal & Exposure Variables

| Variable | Legend |
| --- | --- |
| specie | Animal species handled (cow, goat, pig, etc.) |
| Animal source | Source of animal: 1 = Personal farm, 2 = Bought from market, 3 = Bought from slaughter house |
