# Supplementary material for: Awareness of anthrax disease and the knowledge of its transmission and symtoms identification: A cross sectional study among butchers in Ile-Ife
Source: PLOS Glob Public Health. 2026 Mar 20;6(3):e0005387. doi: 10.1371/journal.pgph.0005387 (PMC13004521; doi:10.1371/journal.pgph.0005387)
Supplement: S3 Table — (DOCX) [file pgph.0005387.s003.docx]

S3 Table: Behavioral & Knowledge Variables

| Variable | Legend |
| --- | --- |
| b1 | Heard about anthrax (1 = Yes, 2= No) |
| b2 | Source of information about anthrax (1 = Friends/ Neighbour, 2=Hospital, 3= Social/Mass media, 4= Local market, 5 = Nil) |
| b3 | Knowledge of transmission of anthrax (1 = Yes, 2= No) |
| b4_1 | Anthrax can be transmitted by eating infected animal (1 = Yes, 2= No) |
| b4_2 | Anthrax can be transmitted by handling infected animal (1 = Yes, 2= No) |
| b4_3 | Anthrax can be transmitted through contaminated soil (1 = Yes, 2= No) |
| c1 | There is relationship between anthrax and sudden death of animal (1 = Yes, 2= No) |
| c2 | Can you identify an animal who has anthrax? (1 = Yes, 2= No) |
| c3_1 | if c2 is yes, symptoms of anthrax- animal die without illness signs (1 = Yes, 2= No) |
| c3_2 | if c2 is yes, symptoms of anthrax- dark unclotted blood flow (1 = Yes, 2= No) |
| c3_3 | if c2 is yes, symptoms of anthrax- carcass not stiff (1 = Yes, 2= No) |
