# Supplementary material for: Awareness of anthrax disease and the knowledge of its transmission and symtoms identification: A cross sectional study among butchers in Ile-Ife
Source: PLOS Glob Public Health. 2026 Mar 20;6(3):e0005387. doi: 10.1371/journal.pgph.0005387 (PMC13004521; doi:10.1371/journal.pgph.0005387)
Supplement: Attachment — Submitted filename: response to reviewers.docx.pdf [file pgph.0005387.s008.pdf]

Manuscript ID: PGPH-D-25-03087

Title: Awareness of Anthrax Disease and the Knowledge of Its Transmission and Symptoms Identification: A Cross-Sectional Study Among Butchers in Ile-Ife

We sincerely thank both reviewers for their insightful and constructive feedback, which has significantly strengthened our manuscript. We have carefully addressed each comment point-by-point, as detailed below. All changes in the revised manuscript are highlighted in yellow for your convenience.

**Reviewer 1**

**Comment 1**: The authors were not consistent in the use of tenses. They should stick to the use of past tenses or continuous tenses. In line 43 they mentioned 'were not aware' but in 45 used 'are aware'. Aware 'of' is more preferable than aware 'about'. They were also not consistent in the use of singular or plural words.

Response: We sincerely thank the reviewer for this detailed observation. The abstract has been thoroughly revised for grammatical consistency. We have standardized all verbs to the past tense, replaced "aware about" with "aware of," and ensured consistent use of plural nouns (e.g., "animals") where appropriate.

**Comment 2**: The authors' conclusion that there was poor awareness is not consistent with the fact that there was no mention of scoring the questionnaire in the methods... Knowledge should be captured both in the methods and results.*

Response: We agree and apologize for this oversight. The methodology section has been expanded to explicitly describe the knowledge scoring system (see Lines 154-158 in the revised manuscript).

A new subsection, "Scoring and Categorization of Knowledge," details that a score was calculated from Section C (10 items), and respondents were categorized as having "Good" or "Poor" knowledge based on the mean score. This categorization and the resulting proportions (47.5% Good, 52.5% Poor) are now clearly presented in the Results section (Lines 198-200).

**Comment 3**: Introduction: Remove the space between the punctuation and the citation... Anthrax is a disease (not an agent) caused by Bacillus anthracis not 'Bacillus Anthrax'.*

Response: Corrected. All in-text citations have been formatted without spaces before the bracket. The organism's name has been corrected to *Bacillus anthracis* throughout the manuscript.

**Comment 4**: Line 75: Change 'buy' with 'but'. Authors should stick to 'human or 'humans'... The transition from one paragraph to the other is not smooth... The statement that the first case of anthrax in Nigeria was in 2023 is a lie!

Response: The typo has been corrected to "but". The term "humans" is now used consistently. The background section has been substantially restructured for better logical flow and to clarify our statement. We have rephrased it to state accurately: "In Nigeria, the first confirmed animal case of the 2023 regional outbreak was reported in July..." to avoid any misinterpretation (Lines 75 -76). The preceding paragraphs have been rewritten to improve transitions.

**Comment 5**: Line 104: The authors seem to have changed from reporting a study to writing a proposal.

Response: We have revised the final paragraph of the Introduction to maintain a consistent narrative focusing on the study's rationale, removing proposal-like language.

**Comment 6**: Lines 105 -- 108 require referencing.

Response: Appropriate citations have been added to support the statements about livestock's economic and socio-cultural role.

**Comment 7**: Lines 111 and 112: Change 'has' to 'have'. Line 118: 'Osun - State, South-Western', should be written 'Osun State, southwestern'. Line 121: Change 'it\'s' to 'its'. Line 125: Change 'accommodates' to 'accommodate'. The authors should draw a map of the area being described.

Response: All grammatical and formatting errors have been corrected. A map of the study area (Ile-Ife, Osun State) has been created and included as Figure 1 in the revised manuscript. The previous Figure 1 (source of information) is now Figure 2.

**Comment 8**: The inclusion and exclusion criteria are confusing... How did the authors identify healthy and sick butchers?

Response: The criteria have been rephrased for clarity. The inclusion criteria now specify "active, licensed commercial butchers operating from a fixed stall/stand." The exclusion criterion regarding illness has been clarified as "Acute illness at the time of interview that impaired the ability to participate," as determined by the researcher's observation and respondent self-report at the point of contact.

**Comment 9**: Lines 140 - 149: The fact that the authors are contemplating calculating sample size and recruiting respondents for a study that is concluded is absurd. Lines 158 and 159: Change 'was' to 'were'.

Response: The text in the Methods section has been changed to the past tense (e.g., "was calculated," "were recruited") to accurately reflect that these steps were completed. The language has been standardized throughout the section.

**Comment 10**: Which random sampling did the authors use? …What was the sampling frame?... The authors did not explain whether and how the questionnaire was validity and reliability tested.

Response: We thank the reviewer for this crucial methodological critique. The "Sampling techniques" subsection has been completely rewritten and expanded (Lines 119-132). We now explicitly detail the two-stage cluster sampling process:

Stage 1: The sampling frame (list of 28 markets from the Ministry of Trade) and the use of computer-generated random numbers to select 10 markets.

Stage 2: The process of obtaining on-site lists of butchers and applying systematic random sampling within each market.

The "Data collection" subsection now includes a detailed description of questionnaire validation, including translation/back-translation, expert review for content validity, and a pilot study (n=45) with Cronbach's alpha ($\alpha$=0.78) to establish reliability (Lines 134-147).

**Comment 11**: Results: Line 167: Change 'questionnaires' to 'questionnaire'... Line 174: Change 'rare' to 'rear'. The authors claimed to have worked with only commercial butchers. How come most of them have become cattle rearers?

Response: "Questionnaires" has been changed to "questionnaire." The typo has been corrected to "rear." We have clarified in Table 1 that the "Specie of livestock" refers to the primary type of

animal they handle/slaughter, not necessarily own. The heading has been amended to "Primary livestock species handled" to avoid confusion.

Comment 12: Discussion: The authors seem not to understand that there is a difference between knowledge and awareness... The authors' claims on knowledge level may not be correct without scoring…

Response: We have revised the Discussion to clearly differentiate between "awareness" (having heard of the disease) and "knowledge" (understanding its specifics). The knowledge claims are now firmly based on the described scoring system. The comparative analysis with Cadmus et al. (2024) has been reviewed and replaced to ensure it aligns appropriately with our new, scored knowledge outcomes.

Comment 13: Limitations: Secondary education is not low level of literacy...

Response: We have removed the phrase "low level of literacy" from the limitations. The relevant limitation now focuses on the potential for "social desirability bias" in self-reported data.

**Reviewer 2**

**Comment 1**: As regards the study design, it would be appropriate to clarify the sampling frame, randomization process, and justify the adequacy of the final sample size.

Response: The sampling methodology has been comprehensively clarified in the revised manuscript (Lines 120-132). The sample size calculation, with an adjustment for non-response, is provided to justify the initial target, and the high response rate (84.4%) is reported to confirm adequacy.

**Comment 2**: No basic inferential analyses (e.g., chi-square tests) were performed to identify factors associated with awareness and knowledge... Chi-square tests and logistic regression would identify high-risk subgroups...

Response: We thank the reviewer for this excellent suggestion, which greatly enhances the public health utility of our findings. We have performed new inferential analyses as recommended:

Association between sociodemographic characteristics and knowledge of Anthrax: Among aware respondents, cross-tabulation (Chi-square) revealed significant associations between good knowledge and higher education, higher income, and sourcing from personal farms (Lines 202-213).

**Comment 3**: The discussion could be strengthened by addressing any potential biases... The paper could explore in greater depth why the awareness was low... the conclusion... could be more specific about what measures should be taken.

Response: The Discussion has been significantly strengthened:

The analysis of why awareness is low has been deepened by integrating findings from the new inferential statistics, discussing how lower education and income create barriers to information access (Lines 254-256).

A dedicated sentence on limitations, including recall and social desirability bias, has been added (Line 270-272).

The Conclusion and Recommendations have been made more specific and actionable. We now recommend: 1) Prioritized, low-literacy health education in markets, 2) Leveraging knowledgeable

farmer-butchers as peer educators, and 3) Integrating zoonosis modules into butcher licensing/training (Lines 280-286).

**Comment 4**: The figures could benefit from more detailed captions.

Response: The captions for both figures (now Figure 1: Study Area Map and Figure 2: Source of Anthrax Information) have been expanded to provide clear, standalone descriptions of what is being shown.

Comment 5: Furthermore, the manuscript contains grammatical errors and spelling mistakes throughout...

Response: The entire manuscript has undergone thorough proofreading and professional language editing to correct grammatical errors, spelling mistakes (e.g., "symptoms" in title, "unclotted blood"), and ensure consistent formatting (e.g., B. anthracis in italics). Unclear and repetitive sentences, particularly in the Background and Discussion, have been rephrased for clarity and conciseness.

We believe the revised manuscript is now substantially improved, methodologically robust, and clearly written. We are grateful for the reviewers' time and expertise, which have been invaluable in enhancing the quality and impact of our work.
