# Supplementary material for: Awareness of anthrax disease and the knowledge of its transmission and symtoms identification: A cross sectional study among butchers in Ile-Ife
Source: PLOS Glob Public Health. 2026 Mar 20;6(3):e0005387. doi: 10.1371/journal.pgph.0005387 (PMC13004521; doi:10.1371/journal.pgph.0005387)
Supplement: Attachment — Submitted filename: Response to reviewer.pdf [file pgph.0005387.s009.pdf]

We sincerely thank the reviewer for these thoughtful and constructive comments, which have significantly improved the quality and rigor of our manuscript. We believe the revised version now presents a clearer, more methodologically sound, and more impactful study. We hope that the changes made meet with your approval and look forward to your response.

Comment 1 (Abstract): "To better frame the study's contribution, the abstract should identify the specific knowledge gap or problem that the research aims to fill."

Response: Thank you for this valuable observation. We have revised the abstract to explicitly state the knowledge gap that necessitated this study.

Comment 2 (Lines 138-139 - Sampling techniques): "This should state: Every k-th butcher was selected, starting from a number chosen by balloting (or computer generated) between 1 and k. The current description ('...starting from a randomly chosen...') is methodologically vague. For clarity and rigor, it is better to specify the exact process."

Response: We appreciate this call for methodological precision. We have revised the sentence to specify the exact process used for selecting the starting point. The revised text now reads:

"Every k-th butcher was selected, starting from a number chosen by balloting between 1 and k."

Comment 3 (Tables): "Please revise the titles for Tables 1, 2, and 3 to be fully self-explanatory. Table titles must be complete and interpretable without reference to the main text. Please expand them to include the 'what' and 'who.' For example, Table 3 should be re-titled to specify the population and location, such as 'Knowledge about identification of anthrax symptoms among butchers interviewed in Ile-Ife, Osun State, Nigeria'.

Response: We thank the reviewer for this important observation regarding table clarity. We have revised all three table titles to be fully self-explanatory and interpretable without reference to the main text. The revised titles are:

Table 1:"Sociodemographic characteristics of butchers interviewed in Ile-Ife, Osun State, Nigeria (N=380)"

Table 2: "Knowledge about transmission of Anthrax among butchers in Ile-Ife, Osun State, Nigeria (n=80)’’

Table 3: "Knowledge about identification of anthrax symptoms among butchers in Ile-Ife, Osun State, Nigeria (n=80)"

Comment 4 (Line 211 - Analysis): "The analysis would be strengthened by using logistic regression to control for confounding factors. The discussion should then be revised to reflect the findings from this adjusted model."

Response: We are grateful for this excellent suggestion, which has substantially strengthened our analysis. We have now conducted a binary logistic regression analysis with overall knowledge (good/poor) as the dependent variable. The independent variables entered into the model were education, income, and source of animals (the variables that showed significant associations in the bivariate analysis).

We have revised the Results section to include these logistic regression findings and updated the Discussion section to reflect the implications of this adjusted analysis. The discussion now emphasizes education as the primary and most robust driver of knowledge, and explains how the logistic regression results provide crucial nuance by revealing that income and animal source were not independent predictors once education was controlled for.

Comment 5 (Line 384 - Reference 14): "Reference 14 journal should be abbreviated."

Response: Thank you for noting this inconsistency. We have corrected Reference 14 by abbreviating the journal title according to standard conventions. The reference now reads:

"Kungu JM, Nsamba P, Wejuli A, Kabasa JD, Bazeyo W. Perceptions and Practices towards Anthrax in Selected Agricultural Communities in Arua District, Uganda. *J Trop Med.* 2020; 2020:1-6. doi:10.1155/2020/9083615"*

We have also verified that all other journal titles in the reference list are appropriately abbreviated in accordance with the journal's style requirements.

Location in revised manuscript: References section, Page 15, Reference 14.

Summary of Changes

| Reviewer Comment | Action Taken | Location in Manuscript |

|-----------------|--------------|-----------------------|

| 1. Abstract - identify knowledge gap | Added sentence specifying the research gap | Abstract, Page 1, Para 1 |

| 2. Sampling techniques - specify random start method | Revised to specify "chosen by balloting" | Methods, Page 5, Lines 138-139 |

| 3. Table titles - make self-explanatory | Expanded all three table titles | Tables 1, 2, 3 (Pages 7-9) |

| 4. Logistic regression analysis | Added regression analysis and revised discussion | Results (Page 10) & Discussion (Page 12) |

| 5. Reference 14 - abbreviate journal title | Corrected to abbreviated format | References, Page 15, Ref 14 |

We sincerely thank the reviewer for these thoughtful and constructive comments, which have significantly improved the quality and rigor of our manuscript. We believe the revised version now presents a clearer, more methodologically sound, and more impactful study. We hope that the changes made meet with your approval and look forward to your response.
