# Supplementary material for: Awareness of anthrax disease and the knowledge of its transmission and symtoms identification: A cross sectional study among butchers in Ile-Ife
Source: PLOS Glob Public Health. 2026 Mar 20;6(3):e0005387. doi: 10.1371/journal.pgph.0005387 (PMC13004521; doi:10.1371/journal.pgph.0005387)
Supplement: Attachment — Submitted filename: PGPH-D-25-03087_reviewer.docx [file pgph.0005387.s005.docx]

I reviewed the manuscript titled ‘Awareness of anthrax disease and the knowledge of its transmission and symptoms identification: a cross sectional study among butchers in Ile-Ife’ and below are my observations:

Abstract

The authors were not consistent in the use of tenses. They should stick to the use of past tenses or continuous tenses. In line 43 they mentioned ‘were not aware’ but in 45 used ‘are aware’. Aware ‘of’ is more preferable than aware ‘about’. They were also not consistent in the use of singular or plural words. For instance, in line 46, they referred to ‘animal’ and then ‘animals’. They can stick to the former as a collective noun or the latter as plural.

The authors’ conclusion that there was poor awareness is not consistent with the fact that there was no mention of scoring the questionnaire in the methods. In addition, the lack of mention of knowledge which is a part of the title in the abstract leaves a major void. Knowledge should be captured both in the methods and results. Otherwise, it should be deleted from the title.

Introduction

Remove the space between the punctuation and the citation in square bracket see lines 63, 79 and 112, for instance. Anthrax is a disease (not an agent) caused by *Bacillus anthracis not ‘*Bacillus Anthrax*’*.

Line 75: Change ‘buy’ with ‘but’.

Authors should stick to ‘human or ‘humans’ (Lines 74 and 76).

The transition from one paragraph to the other is not smooth. For instance, lines 79 and 80 do not have any connection. In fact paragraph 4 has no place in its current position in the background of the study. The first sentence of paragraph 5 makes no sense and should be recast.

The statement that the first case of anthrax in Nigeria was in 2023 is a lie!

The authors should make use of the correct words: For instance, ‘body openings’ should be referred to as ‘body orifices’.

Line 104: The authors seem to have changed from reporting a study to writing a proposal.

Lines 105 – 108 require referencing.

Lines 111 and 112: Change ‘has’ to ‘have’.

Line 118: ‘Osun - State, South-Western’, should be written ‘Osun State, southwestern’.

Line 121: Change ‘it's’ to ‘its’.

Line 125: Change ‘accommodates’ to ‘accommodate’.

The authors should draw a map of the area being described.

Line 134: Change ‘accommodates’ to ‘accommodate’.

The inclusion and exclusion criteria are confusing: ‘Butchers who were standing were included’. Does this mean that those butchers who were sitting were excluded? ‘Butchers who were sick were excluded’, therefore only the healthy were included. How did the authors identify healthy and sick butchers? What was the reason for the chosen criteria?

Lines 140 - 149: The fact that the authors are contemplating calculating sample size and recruiting respondents for a study that is concluded is absurd.

Lines 158 and 159: Change ‘was’ to ‘were’.

Line 161: Which random sampling did the authors use to select the butchers? They should be specific in explaining how the sampling method used was applied in the work.

The sampling was not well explained. The authors claimed to have used multi-stage sampling technique. What was the sampling frame in each stage and how were they prepared or accessed?

The authors did not explain whether and how the questionnaire was validity and reliability tested. Was there a pilot study and how was it conducted? Without these, the instrument is not valid for a meaningful research. How many sections did the instrument contain and how many questions were there in each section? Was the questionnaire scored and how was it scored? Without scoring the questionnaire, the authors cannot determine the levels of awareness or knowledge.

Results

Line 167: Change ‘questionnaires’ to ‘questionnaire’ because only one questionnaire was used for the study.

Line 174: Change ‘rare’ to ‘rear’. The authors claimed to have worked with only commercial butchers. How come most of them have become cattle rearers?

Table 1: Change ‘live stock’ to ‘livestock’.

Line 180 – 181. Recast the sentence.

Discussion

The authors seem not to understand that there is a difference between knowledge and awareness, Therefore, it may be worthwhile to define both words in the work. The authors’ claims on knowledge level may not be correct without scoring knowledge to determine the level among the participants. Comparisons made with some author, example Cadmus et al. (2024) do not rhyme.

Limitations

Secondary education is not low level of literacy. Therefore, an area that has more than 70% of the population that attained secondary education cannot be regarded as having low level of literacy.
