# Supplementary material for: Awareness of anthrax disease and the knowledge of its transmission and symtoms identification: A cross sectional study among butchers in Ile-Ife
Source: PLOS Glob Public Health. 2026 Mar 20;6(3):e0005387. doi: 10.1371/journal.pgph.0005387 (PMC13004521; doi:10.1371/journal.pgph.0005387)
Supplement: Attachment — Submitted filename: PGPH _D_25_03087_reviewer report.docx [file pgph.0005387.s007.docx]

PGPH-D-25-03087R1

I reviewed the manuscript titled: “Awareness of anthrax disease and the knowledge of its transmission and symptoms identification: a cross sectional study among butchers in Ile-Ife”.

The study effectively documents a severe and highly specific public health vulnerability—low anthrax awareness and knowledge among Nigerian butchers—a critical frontline group. Its principal contribution lies in precisely characterizing this gap, identifying high-risk subgroups, and highlighting reliance on informal information networks. The findings are immediately actionable for designing targeted, community-based interventions and strengthening surveillance in urban meat supply chains. Although the manuscript has been greatly improved, there are few issues that need to be addressed.

Abstract: **To better frame the study's contribution, the abstract should identify the specific knowledge gap or problem that the research aims to fill.**

Line 138 - 139: This should state: Every k-th butcher was selected, starting from a number chosen by balloting (or computer generated) between 1 and k.

**The current description ('…**starting from a **randomly** chosen…**’) is methodologically vague. For clarity and rigor, it is better to specify the exact process.**

Tables: **Please revise the titles for Tables 1, 2, and 3 to be fully self-explanatory. Table titles must be complete and interpretable without reference to the main text. Please expand them to include the 'what' and 'who.' For example, Table 3 should be re-titled to specify the population and location, such as ‘Knowledge about identification of anthrax symptoms among butchers interviewed in Ile-Ife, Osun State, Nigeria’.**

Line 211: **The analysis would be strengthened by using logistic regression to control for confounding factors. The discussion should then be revised to reflect the findings from this adjusted model.**

Line 384: Reference 14 journal should be abbreviated.
